# Commentary: Towards a unifying mechanism for cancelling movements

**DOI:** 10.3389/fpsyg.2019.00879

**Published:** 2019-05-07

**Authors:** Olivier A. Coubard

**Affiliations:** The Neuropsychological Laboratory, CNS-Fed, Paris, France

**Keywords:** movement suppression, decision cancellation, stopping, inhibition, theory

Recently, Noorani (2017) suggested a mechanism for stopping and stillness as part of a themed issue about movement suppression by Carpenter and Noorani (2017). Motor control is made up of actions such as preventing and initiating movement or stopping initiated movement. How animals and humans stop or stay still, has been subjected to the development of experimental tasks, theoretical models, neural exploration, and hypotheses about the relationships between tasks, models and neural evidence. Noorani reviews four paradigms in which cancellation of an impending eye movement decision is required: countermand or stop signal, go/no-go, anti-saccade, Wheeless, and redirect tasks. The paradigms are read through the lens of Bayesian decision theories advocating the existence of Go and Stop decision signals for movement initiation and stopping, respectively. Neurophysiological and brain imaging studies have suggested that there may indeed exist independent and interacting Go and Stop neural units. The author opens the question of whether the brain contains several Stop units or a single one acting in the different tasks. His conclusion is that a unifying Stop mechanism races against distinct Go units, and that the difference between tasks only relies on the amount of processing required to evaluate the Stop signal (Noorani, 2017).

Noorani (2017) opinion is interesting as it connects perfectly to earlier published models. The idea of a unifying mechanism for cancelling movements, together with that of functionally linked groups of neurons in distributed regions of the brain to ensure this process, has been developed in the Threshold Interval Modulation with Early Release-Rate of rIse Deviation with Early Release (TIMER-RIDER) model by Coubard (2012) (Figure 1A). In this model based on human physiology and chemistry, the brain is permanently animated by two main streams, excitatory glutamatergic, and inhibitory gamma amino-butyric acidergic, in an estimated 60–40% proportion. A small part of activity is devoted to modulation through neurotransmitters like noradrenaline and serotonin. Rather than racing, both streams work in subtle harmony to allow the emergence of adaptive behaviors throughout phylogenesis and ontogenesis (Coubard, 2016). The TIMER-RIDER model has three important features. First, the stream of inhibitory mechanisms is embodied by an attention-inhibition network (AIN), in which a unique inhibition process acts to cancel movements. Second, the AIN is global and well distributed from brainstem to cortex. A group of AIN neurons can inhibit distinct Go units. Reversely, distributed linked groups of AIN neurons can inhibit a single Go unit. Third, the inhibition process is early, controlling upstream excitatory units for movement (Coubard, 2012). Noorani (2017) opinion is also reminiscent of the model by Kenemans (2015) describing reactive inhibition taking roots in the dorsal-medial prefrontal cortex, which generalizes to situations in which behavioral interrupt is invoked by the salience of distractors (this activity being akin to TIMER modulation), and proactive inhibition as originating from ventral-lateral prefrontal cortex, which potentiates inhibitory sensorimotor connections (resembling RIDER modulation).

**Figure 1:**
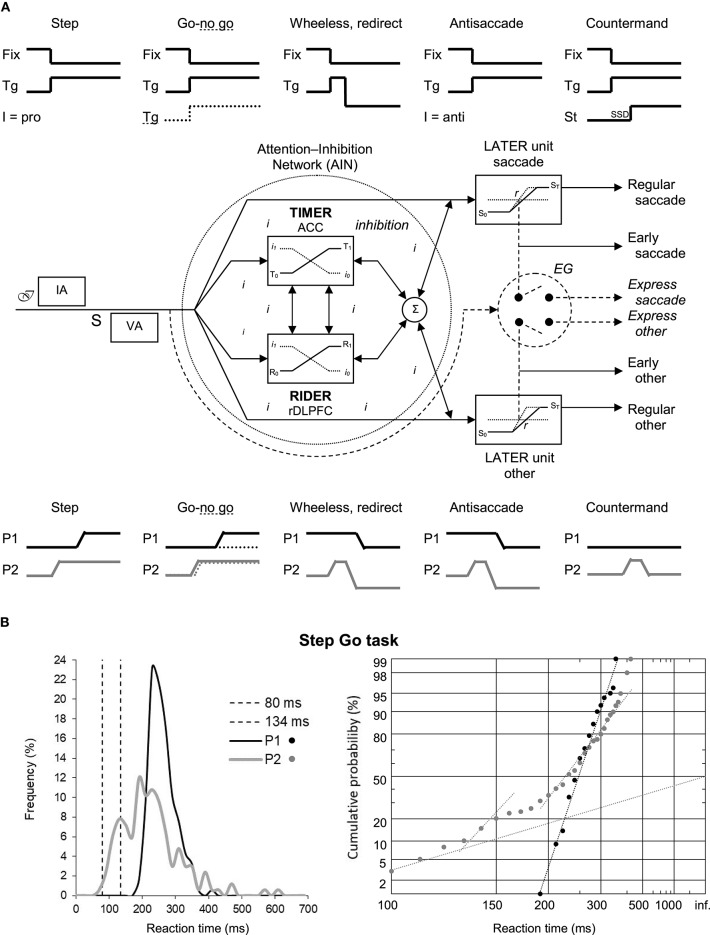
**(A)** The Threshold Interval Modulation with Early Release-Rate of rIse Deviation with Early Release (TIMER-RIDER) model explains the different paradigms of movement cancellation or not. (**A**-up). We show the stimuli signals for fixation point (Fix) and lateral target (Tg) in the Step, Go-no go, Wheeless or redirect, Antisaccade, and Countermand eye movement paradigms. In Step and Antisaccade paradigms, the visual stimulation is the same but the instruction (I) calls for a saccade in the direction of the target (pro) or in the opposite direction (anti), respectively. In Go-no go paradigm, the target takes some color (full line) or another one (dotted line) calling for a prosaccade or nothing, respectively. In Countermand paradigm, a Stop (St) signal occurring after a Stop Signal Delay (SSD) calls for movement stopping. (**A**-middle). The TIMER-RIDER model. After psychological processes involving the intention of doing the task (ℐ), instruction analysis (IA), the appearance of stimuli (S), visual analysis (VA), an early stream of inhibitory mechanisms takes place within a global and distributed attention-inhibition network (AIN) where a single inhibition process (*i*) determines either upcoming movement by the Go (linear approach to threshold with ergodic rate, LATER) units, or movement suppression. Distinct LATER units exist for different types of movement (saccade, other). The modulation is ensured by TIMER and/or RIDER to influence respectively the distance between initial (S_0_) and final (S_T_) thresholds and/or the rate of rise of the decisional signal within the LATER units. To date, the anterior cingulate cortex (ACC) and the right dorsolateral prefrontal cortex (rDLPFC) have been suggested to participate in TIMER and RIDER modulation, respectively, but other brain areas for modulation are not excluded. Early movements emerge under optimal TIMER and/or RIDER activity resulting in minimal inhibition. A shorter route enables to bypass the AIN, resulting in express saccades or other movements. (**A**-bottom). We illustrate in each paradigm the corresponding saccade signals of participant 1 (P1) having a strong AIN and of participant 2 (P2) having a weak one. P1 and P2 are healthy women aged 81 and 77 years, respectively, living autonomously at home. Their common characteristic is to have fallen more than the average in the last year for a yet unknown reason at the time of the recording. **(B)** Results of P1 and P2 in a simple Step Go task. (**B**-left). Frequency histogram (in %) as a function of reaction times (in ms) for saccades between 0 and 700 ms. Bin width is 20 ms. Vertical dotted lines indicate the standard limits of express saccades: 80 and 134 ms. (**B**-right). Corresponding reciprobit plots of Cumulative probability (in %) as a function of reaction times (in ms) for saccades between 100 and infinite. *N* = 133–185 in frequency histograms; *N* = 130–185 in reciprobit plots. Data taken from Coubard (2012).

With these models in mind, some aspects of Noorani (2017) opinion may still need improvement to be physiologically implementable. Minor inaccuracies concern the suggestion that the neocortex might exclusively process colors or spatially invert target locations. This obsolete Jacksonian neurology was recently deconstructed (Chang et al., 2016; Hall and Colby, 2016; Herman and Krauzlis, 2017). More substantial, Noorani (2017) comprehension and description of brain functioning is “taskomorphic”—a neologism I introduce here to mean that the brain, according to the author, may work as the tasks are built. Accordingly, a task containing Go and Stop signals (e.g., countermand task) yields brain implementation involving Go and Stop neural units, whereas a task containing a Go signal (e.g., simple step task) is assumed to involve a Go neural unit only. This common pitfall in psychology takes root in the assumption that human achievements tell us something about human brain functioning. Another bias is the difficulty by Noorani to disentangle the primary mechanism of stopping and stillness from the mechanisms of modulating stopping and stillness. Neurophysiological studies commonly tend to mix the respective roles of brain areas due to their close vicinity in either rodents, Felinae or primates. For instance, since Moschovakis et al. (1996), the respective roles of frontal eye field and prefrontal cortex in saccade suppression have regularly been confused (e.g., Hanes et al., 1998). Following Stuphorn and Schall (2006), the suggestion by Noorani (2017) that the supplementary eye field suppresses saccades remains to be demonstrated.

TIMER-RIDER model has several advantages. It explains normal and pathological behaviors in paradigms in which movement cancellation is required or not (Figure 1A). The model is also compatible with old and recent neurophysiological and brain imaging findings. In line with the second feature (i.e., global and distributed AIN), the raphe nuclei inhibit multiple saccade premotor generators, whilst the dorsolateral prefrontal cortex, substancia nigra pars reticulate, and rostral superior colliculus (SC) inhibit only the caudal SC (Goldman and Nauta, 1976; Hikosaka and Wurtz, 1983; Munoz and Wurtz, 1993; Coubard, 2011). Consistent with the third feature (i.e., early inhibition process), studies have provided neural evidence of a rapid or early process (Stanford et al., 2010; Salinas and Stanford, 2013; Schmidt et al., 2013). This characteristic also sheds light on brain imaging studies suggesting ultra-rapid cognitive processing, which can be read as early inhibitory processing in a detection task (Thorpe et al., 1996). Importantly, the inhibitory stream is continuously active even in tasks without a Stop signal. Let us consider two healthy participants, P1 with a strong AIN and P2 with a weak one, performing a simple step (Go) task (Figure 1B). P1 exhibits unimodal distribution in a traditional plot and a straight line in a reciprobit plot, meaning that a single saccadic population can be modeled by only two parameters. In contrast, P2 performance yields multimodal distribution and three subtypes of recinormal distributions of so-called main, express and early saccades requiring at least five parameters. In both participants, excitatory (Go) and inhibitory (Stop) mechanisms are permanently present, whilst inhibitory mechanisms are only visible in P2 due to their failure. In other words, what is invisible to the eye does not mean it does not exist.

## Author Contributions

The author confirms being the sole contributor of this work and has approved it for publication.

### Conflict of Interest Statement

The author declares that the research was conducted in the absence of any commercial or financial relationships that could be construed as a potential conflict of interest.
